# medspacyV: a graphical user interface for the open source medspaCy natural language processing package

**DOI:** 10.1093/jamiaopen/ooaf094

**Published:** 2025-08-23

**Authors:** Bharath Velamala, Elham Sagheb Hossein Pour, Michael Lin, Jungwei Wilfred Fan

**Affiliations:** Department of Artificial Intelligence and Informatics, Mayo Clinic, Rochester, MN, 55905, United States; Center for Clinical and Translational Science, Mayo Clinic, Rochester, MN, 55905, United States; College of Information Science, the University of Arizona, Tucson, AZ, 85721, United States; Department of Artificial Intelligence and Informatics, Mayo Clinic, Rochester, MN, 55905, United States; Center for Clinical and Translational Science, Mayo Clinic, Rochester, MN, 55905, United States; Department of Artificial Intelligence and Informatics, Mayo Clinic, Rochester, MN, 55905, United States; Center for Clinical and Translational Science, Mayo Clinic, Rochester, MN, 55905, United States; Department of Quantitative Health Sciences, Mayo Clinic, Rochester, MN, 55905, United States

**Keywords:** natural language processing, user-computer interface, software design

## Abstract

**Objectives:**

To enable users with modest technical background to perform biomedical natural language processing (NLP).

**Materials and Methods:**

We developed medspacyV using the Python graphical programming tkinter library, following the model-view-controller (MVC) design pattern. The interface wraps around a rule-based pipeline for sentence splitting, section segmentation, concept identification, and negation detection.

**Results:**

The primary window allows the user to configure the project and NLP rules, execute the pipeline, and save the outputs into a table. A separate annotation viewer window can be launched to inspect the immediate or previous NLP outputs.

**Discussion:**

We developed medspacyV with three rationales: controllability, explainability, and economy. The rule-based approach is sufficient for many NLP use cases.

**Conclusion:**

The medspacyV program is publicly available at https://github.com/medspacy/medspacyV, targeting use by healthcare professionals and researchers in their NLP projects.

## Objectives

The program medspacyV was developed to offer a user-friendly graphical interface for configuring and running a rule-based natural language processing (NLP) pipeline, based on the open-source package medspaCy.^1^ The tool allows users with modest technical training to define and execute any NLP project involving the fundamental tasks of sentence splitting, section segmentation, concept identification, and negation detection.

## Background and significance

A wealth of data involved in clinical practice and research is unstructured, including medical notes and scientific literature. Due to the known challenges around domain-specific vocabularies, meanings, and styles, biomedical NLP remains a vibrant area of research over the past decades. Hundreds of tools have been developed to assist in tasks ranging from generically defined biomedical information extraction and normalization to specific applications such as adverse event detection and trial eligibility screening.^2^ Despite the overwhelming success of large language models (LLMs) in numerous applications, they can be less favored when the cost, fidelity, and consistency issues are put under scrutiny.^3^ In contrast, traditional rule-based NLP is sufficient in many well-defined, simple information extraction tasks, while offering transparency, full control, and economical computing that is generally performed within a secure local environment. Or combining the best of both worlds, a potential hybrid approach is to first leverage LLMs for discovering comprehensive patterns, which can then be loaded into a rule-executing engine for secure and economical computation.

Therefore, we believe there is significant value in making such a rule-authoring and/or rule-executing tool accessible for the broad biomedical NLP user community. Instead of reinventing the wheel, we came across medspaCy as an ideal backend to bootstrap from. The medspaCy^1^ is an extensible, open-source biomedical NLP library based on the spaCy framework implemented in Python. It offers rich functionality via several customizable core components: sentence splitting, section segmentation, concept identification, and negation detection. Numerous projects have leveraged medspaCy, such as extraction of housing status^4^ and patient-reported outcomes in chiropractic notes.^5^ Although most of the components are rule-based, writing a Python script to link them still poses a hurdle to non-programmers. To eliminate this hurdle, we developed a user-friendly graphical desktop application, medspacyV(isual), which avails medspaCy’s key components for non-technical users to conduct a variety of biomedical NLP projects.

## Materials and methods

The medspacyV application is built using *tkinter* (https://docs.python.org/3/library/tkinter.html), a well-established Python library for graphical user interface (GUI). We followed the Model-View-Controller (MVC) design pattern^6^ in structuring the code base, which offers the advantages of maintainability and scalability. For the backend, we streamlined a series of fully controllable rule-based medspaCy components:

1. Sentence splitter preloaded with the RuSH^7^ segmentation rules
2. Section detector based on medspaCy’s section ontology, which can also be customized with institution-specific section headers
3. Concept identifier that allows the user to define terms or regular expressions for concept extraction and normalization
4. Negation detector powered by the ConText^8^ algorithm to identify negation, certainty, and experiencer (patient vs. family member)

For the frontend interface, medspacyV renders standard look-and-feel windows centered around the workspace (one active project at a time). The primary window allows the user to specify the input/output folders, and most importantly the configurable rules for each of the NLP components. Upon clicking to edit each component’s rules, a corresponding application will be opened: for concepts it is an Excel worksheet and for the other components it is Notepad plaintext. To allow for downstream analyses and integration with other data sources, a tabular output is generated upon completing the processing of a document set, with each row enclosing an identified concept and its attributes. Another notable feature is the Annotation Viewer, a separate window that can be launched to inspect the NLP-identified concepts and attributes including the negation indicator and host section.

For proper functioning, Python 3.8.10 or higher version is recommended; a list of required modules is provided in Table 1.

**Table 1.** Required Python modules in medspacyV.

Library name	Minimum version
medspacy	1.2.0
numpy	1.24.4
openpyxl	3.1.3
pandas	2.0.3
Pillow	10.4.0
pyinstaller	5.13.2
py-splash	0.4.5
Regex	2024.5.15
Spacy	3.5.4

## Results

**Figure 1.**
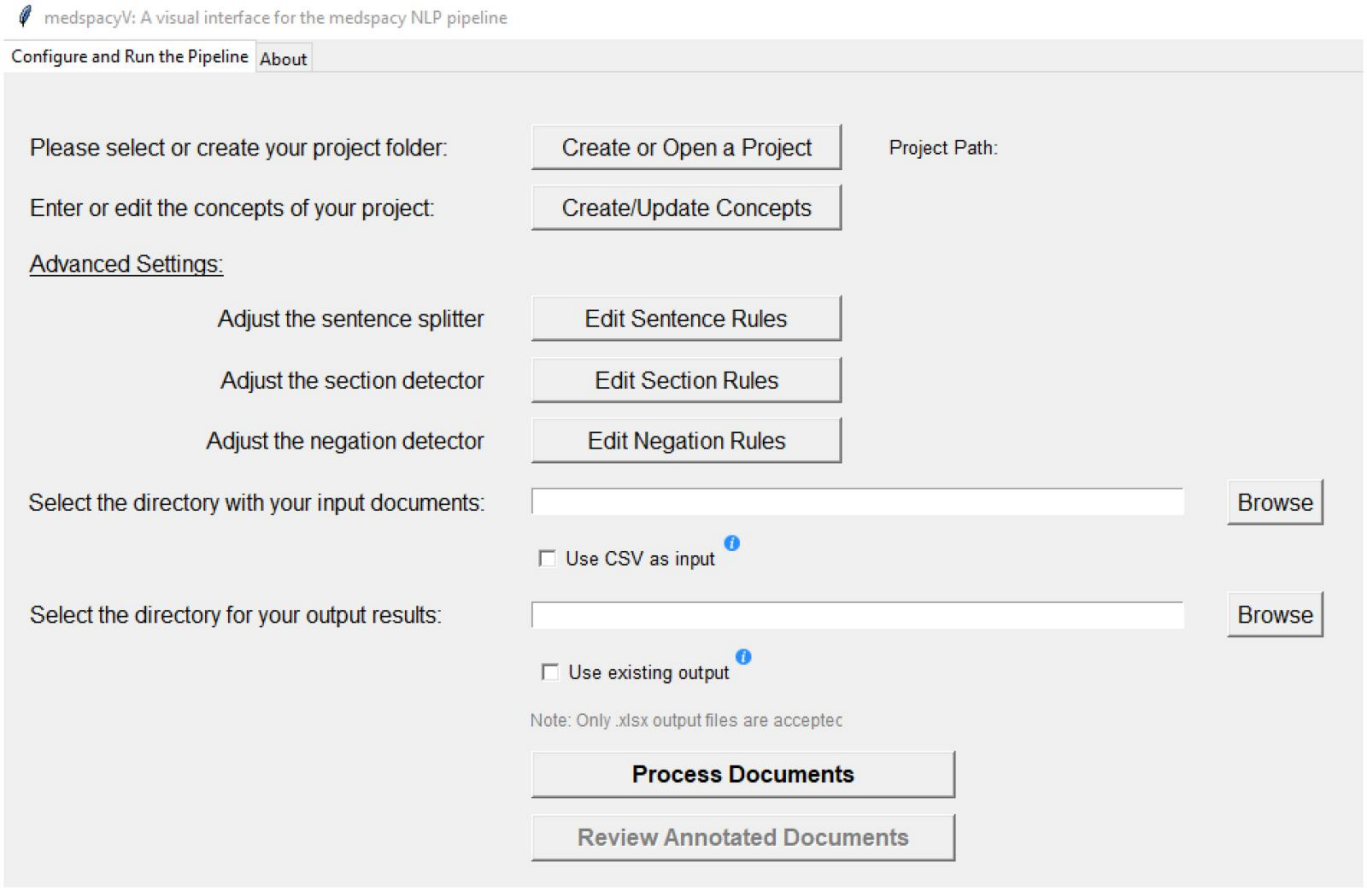
The primary window of medspacyV, for specifying the project directory, NLP component rules, input and output paths, and executing the pipeline.

**Figure 2.**
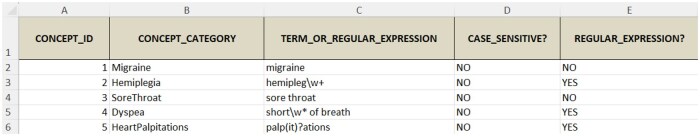
An example configuration of concept rules, which specify the normalization labels, synonymous terms and patterns, as well as the switches for case sensitivity and use of regular expression.

The primary window of medspacyV is shown in Figure 1. In a general scenario, the user would start by creating and specifying the project folder that serves as the workspace for a corresponding NLP use case. An essential subfolder under the project is “resources,” which contains the rule files used by each of the NLP components. For convenience, we have shared a template project on medspacyV’s public release page for users to modify from. Once the project folder is set, the rule files will be automatically loaded and be editable. For most use cases, the main component to work out is concept identification and normalization. As an example, Figure 2 shows how the editable concept rules look like when opened in the worksheet. The user can determine whether a rule pattern is term match only or by regular expression, as well as case sensitivity, which is useful when strict all-caps acronym match is needed. The column “CONCEPT_CATEGORY” serves as the normalization label that all the synonymous terms and patterns of a concept should be mapped to. Besides, the sentence splitter, section identifier, and negation detector (ConText algorithm) all work out of the box by using domain-adapted rules from the original medspaCy package. They can be further refined but their rule authoring requires more NLP knowledge and therefore are indicated under “Advanced Settings.”

**Figure 3.**
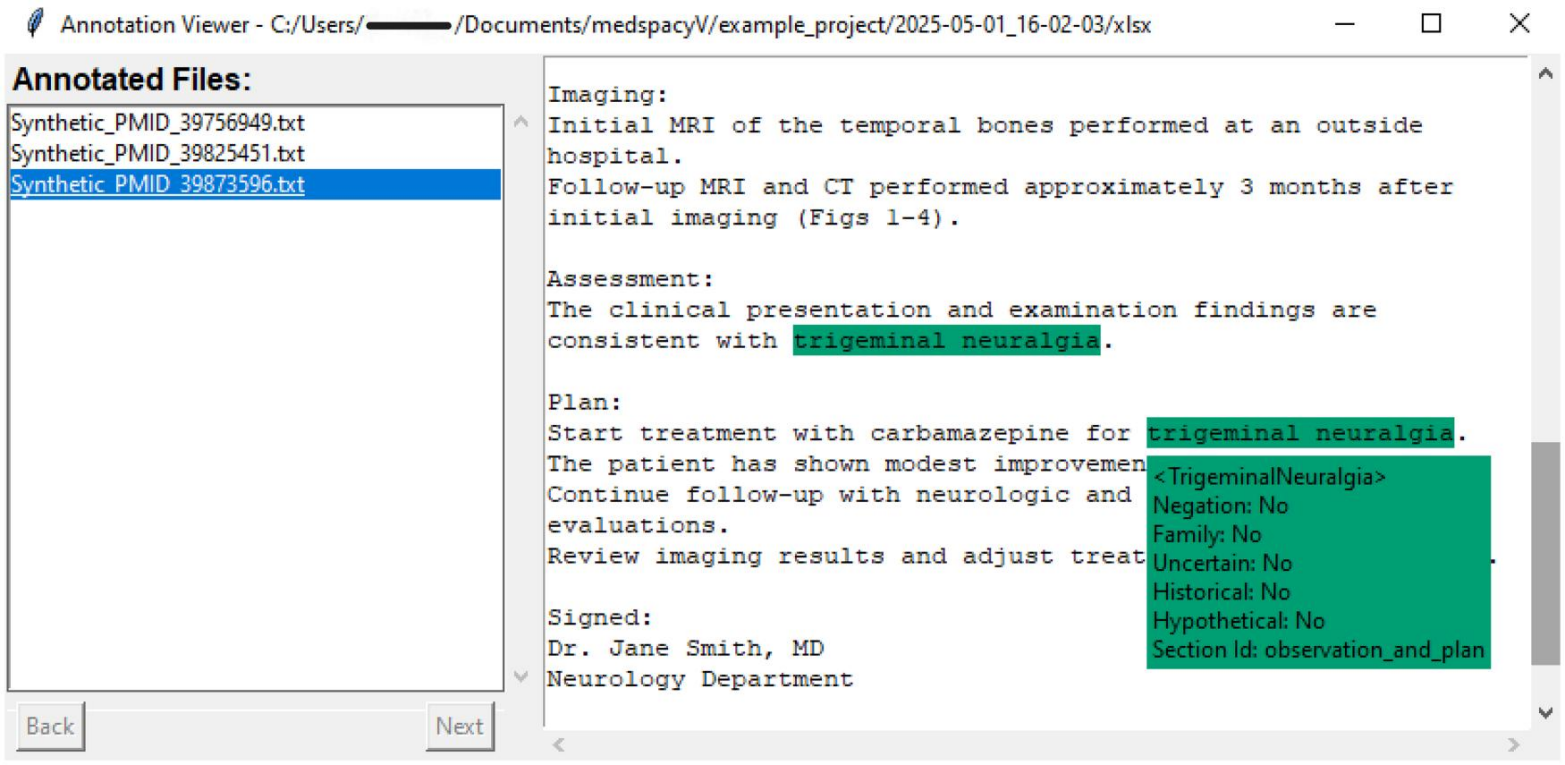
The annotation viewing window of medspacyV, for selecting which document to inspect and browsing the NLP-identified concepts and their attributes.

After the notes are processed, the default output is a table (available in CSV and XLSX) of the identified concepts (see eg, Table 2) saved under the project folder. By clicking the button “Review Annotated Documents” on the primary window, the user can inspect the identified concepts and their attributes. For example, Figure 3 shows two identified occurrences of the concept Trigeminal Neuralgia in the note. A dynamic pop-up box is triggered by hovering the mouse cursor over a highlighted extraction, displaying the ConText binary flags including negation and the normalized label of the detected host section (eg, “observation_and_plan” in this case). Moreover, medspacyV allows visualizing results from any earlier processed notes, as long as the folders containing the output annotations and the corresponding original input documents are specified properly. With feedback from a pilot user, we have also implemented accepting CSV (comma-separated values) as input, which embeds the clinical notes in one designated column along with additional metadata (eg, demographics) in other columns to facilitate convenient linkage for downstream analyses.

**Table 2.** Example of tabular outputs from medspacyV (rows and columns are transposed for readability), showing two extracted concepts.

doc_name	Synthetic_PMID_39873596.txt	Synthetic_PMID_39873596.txt
concept (normalized label)	Pain	TrigeminalNeuralgia
matched_text	pain	trigeminal neuralgia
concept_start	75	1542
concept_end	79	1562
sentence	Left-sided facial pain for 5 weeks.	Start treatment with carbamazepine for trigeminal neuralgia.
sentence_start	57	1503
sentence_end	94	1563
section_id (normalized label)	chief_complaint	observation_and_plan
matched_section_header	Chief Complaint	Assessment
is_negated	FALSE	FALSE
is_family	FALSE	FALSE
is_uncertain	FALSE	FALSE
is_historical	FALSE	FALSE
is_hypothetical	FALSE	FALSE

## Discussion

We developed and shared the medspacyV application to make a rule-based biomedical NLP pipeline more accessible to non-technical users. The underlying rationales for medspacyV are controllability, explainability, and economy. For each pipeline component, we expose the rules that control the processing behavior to be completely editable by the user. As a result, the processing behaviors are also explainable in a what-you-see-is-what-you-get manner. Once set up, the GUI is intuitive to work with and runs on a single machine, which offers economical, lightweight computing and security without sending any data outside the firewall or incurring any metered services. For usability, self-explaining hints and instructions are displayed on the interface, as well as event-triggered (and logged) error messages to aid both interactive and offline troubleshooting.

Both biomedical researchers with limited programming background and analysts with moderate technical experience can benefit from medspacyV. For example, our pilot user was a senior statistical programmer supporting a clinical informatics core that needs a handy NLP tool to obtain phenotype information not available through ICD codes or other structured data. The useful scenario especially applies to rare or under-coded conditions (eg, short telomere syndrome) where the concept synonyms also tend to be well defined. The user appreciated the intuitive frontend of medspacyV that keeps only the essential functions and allows authoring rules by commodity applications like Excel. Moreover, the user highlighted the importance of having the preloaded domain-specific ConText algorithm, which effectively mitigates false positives by detecting negation or family history.

There have been various GUI tools offered to the biomedical NLP community, mostly Java-based. Two of the representative tools, cTAKES^9^ and CLAMP,^10^ are both built upon the UIMA framework and allow incorporating rule-based components by users. While accommodating comprehensive tasks, they are either not fully open-source or impose a steep learning curve in setup (eg, require knowledge of using an integrated development environment). As Python has become the mainstream programming language for data science, the NLP community also embraces its advantages of having a large development community and resourceful libraries. There are GUI tools for NLP in Python (eg, NLTK^11^ and Prodigy^12^), but they are either not open-source or not geared for biomedical text. Therefore, we considered it worth creating an open-source GUI application on top of medspaCy, which is backed by the Python community and already has domain-calibrated components including RuSH^7^ and ConText.^8^

Rule-based NLP has shown its limitations over the years, and the creation of medspacyV is by no means an advocacy for retrograde movement. Given the wide range of complexities in practice, we advocate that rule-based NLP is still a good fit for many projects that would not require sophisticated technology. Or, as alluded to in our introduction, a rule-based pipeline can serve as a later stage for cost-effective implementation by executing the rich patterns discovered and relayed from modern tools like LLMs. Another point to clarify is that we do not claim medspacyV to be hurdle-free or frictionless. It is known that writing regular expressions or modifying advanced rules like those in the ConText algorithms still demands higher technical literacy. However, we expect medspacyV to be mostly self-service ready, or at least ready to be adopted with modest technical support. Lastly, by sharing as an open-source project it is anticipated that the broader NLP developer’s community will be able to take over the continuing improvements of usability and performance.

While the original medspaCy package offers neat visualizing functions such as color-tagging entities and arch-pointing context modifiers to their host entity, we prioritized offering a basic interface (ie, project configuration) along with visual features that were most technically feasible in *tkinter* for displaying the NLP results. Given that the project is now available to the broad open-source community, we do hope future contributors will add even polished visualization to fully realize the strength of medspaCy, especially for advanced users to troubleshoot and enhance rule performance (eg, intermediate checkpoints for debugging why certain desirable output does not come out).

Additional enhancements around usability and functionality have been identified for future work. The pop-up windows for modifying sentence and negation rules are still barebone text editors in this initial release. A more user-friendly interface is desirable for assisting in the edits or debugging of those complex rules. Although medspacyV is intended to be lightweight, it is possible to further increase the processing throughput by distributing the job to multiple cores even on a single machine. We believe that single-span concept extraction is generally adequate and can be postprocessed to form complex concepts. However, there can be benefits to implement generic support for creating rules of multi-span, post-coordinated concepts in the future.

## Conclusion

We developed medspacyV, a self-contained and user-friendly desktop application for biomedical NLP. The rule-based approach ensures transparency, ease of debugging, and flexibility in modifications. It can take a set of text files or a CSV file with embedded notes as input. The ability to review both current and past results can facilitate A/B testing, while the tabular outputs enable integration with downstream analytics. The tool is made available to the public, with the goal of making NLP more accessible and cost-effective for healthcare professionals and researchers.

## Data Availability

The source codes and instructions are available at https://github.com/medspacy/medspacyV, which also includes an executable that can run in Windows environment. The Python *tkinter* library is not bound to any specific operating system. However, at this point we have only thoroughly tested the compilation and functionality in Windows.
